# Book Review: Extended Conceptual Metaphor Theory

**DOI:** 10.3389/fpsyg.2020.01513

**Published:** 2020-07-31

**Authors:** Xiaoming Dong, Manjiang Duan

**Affiliations:** ^1^Foreign Languages Department, Harbin Engineering University, Harbin, China; ^2^School of Marxism, Mudanjiang Normal University, Mudanjiang, China

**Keywords:** conceptual metaphor theory, extended conceptual metaphor theory, literal vs. non-literal meaning, levels of conceptual structure, context

*Metaphors We Live By*, in which conceptual metaphor theory (CMT) was proposed by Lakoff and Johnson, marked the beginning of systematic studies of metaphor in cognitive linguistics. Zoltán Kövecses' new monograph *Extended Conceptual Metaphor Theory* offers an approach that updates CMT by elucidating many issues that researchers have raised against the theory.

The book consists of eight chapters, with the first being the starting point at which the “standard” version of CMT is introduced, including the views in the pioneering work of *Metaphors We Live By* and the works that confirmed, added to, and also modified the original ideas. The subsequent five chapters characterize the new view as “extended CMT,” each beginning with a thought-provoking alternative question that responds to one issue with the “standard” CMT. Chapter 2, “The Abstract Understood Figuratively, the Concrete Understood Literally, but the Concrete Understood Figuratively?,” responds to the idea that there is such a thing as literal meaning by focusing on the assumption that there may be no literal language at all. The author holds that both concrete and abstract concepts have embodied content ontology and figurative construal and that we can profile the ontology part in some cases and the figuratively construed part in others. Chapter 3, “Direct or Indirect Emergence?,” responds to the debate concerning whether the primary metaphor that is the foundation of CMT emerges directly or through a metonymic stage. The author illustrates the claim that metonymies are, to some degree, more primary than primary metaphors. It is suggested that correlation-based metaphors emerge from frame-like mental representations through a metonymic stage. Chapter 4, “Domain, Schema, Frames, or Space?,” responds to the difficulty in identifying appropriate conceptual structures to participate in the formation of conceptual metaphors. By proposing the “multilevel view of conceptual metaphor,” the author argues that each conceptual metaphor is characterized by four levels, with the highest being that of image schemas, the lowest, that of mental spaces, and in between, that of domains and that of frames. Chapter 5, “Conceptual or Contextual?,” addresses the neglect of context within CMT. The author elucidates the assumption that conceptual metaphors are not simply conceptual but are necessarily contextual by drawing heavily on his 2015 book *Where Metaphors Come From*. Chapter 6, “Offline or Online?,” responds to CMT's inability to account for meaning in actual occurrences of metaphorical language in real discourse by explicating the assumption that conceptual metaphor is both an offline and online phenomenon simultaneously. These five main chapters are followed by two integrative summary chapters. The former addresses the components of an emerging new theory and sketches its general framework, and the latter assesses the responses to the five questions discussed above, together with a rough comparison of this newly proposed paradigm with its sister theory, i.e., the view of metaphor as dynamic systems proposed by Gibbs (2017).

As a forceful attempt to extend CMT, the value of the book lies in the course of constructing the process model for conceptual metaphor. Firstly, it innovatively proposes that both concrete and abstract concepts comprise embodied content ontology and figurative construal but differ in proportion. Secondly, the author agrees on the schematic hierarchy of conceptual structures and creatively complements the hierarchy with the mental space level existing at the lowest level of the hierarchy. This view efficiently expounds on the nature of conceptual metaphor as being both offline and online, which remedies the central limitation of CMT that it takes metaphor as relatively static cognition. Another merit is the systemization of the author's former view of context on metaphor (Kövecses, 2015) as well as its comparison with other popular views, especially Gibbs' view of the dynamic systems model, in accounting for metaphorical creativity and context-sensitivity.

Nevertheless, there remain some aspects worth Kövecses' further elaboration. First, although the dynamic nature of metaphor is emphasized in extended CMT, it is not clearly shown in the model (p. 167). In our opinion, the dynamicity of metaphor conceptualization may be more clearly delineated in the model if the temporal sequence of metaphor understanding is taken into account in addition to the logical sequence. Second, there is a lack of communication between different disciplines. Although extended CMT reflects mental processes as part of a psychologically realistic model of metaphor, there appears little evidence through psychological or psycholinguistic experiments. In addition, the author aims to present a theory of metaphor that can be expressed by language in general; however, in actual fact, the only language used to show how the model works is English. It could make interesting predictions as regards what typologically different languages have in common as well as how they differ.

Put at its briefest, this book is an impressive undertaking in its breadth, depth, and insight that readers will look to as an authoritative source on many respects of CMT. This improved version of CMT will enrich the field of study in areas ranging from metaphorical cognition to literary research. For example, researchers can find evidence from other modalities apart from linguistic evidence to support the schematicity hierarchy, and they can also integrate an “interactional turn” in the study of metaphor. Indeed, recent developments in these areas are starting to deepen aspects related to these issues.

## Author Contributions

All authors listed have made a substantial, direct and intellectual contribution to the work and approved it for publication. XD and MD chose the book together. XD wrote the review. MD provided valuable ideas for the drafts.

## Conflict of Interest

The authors declare that the research was conducted in the absence of any commercial or financial relationships that could be construed as a potential conflict of interest.
